# Inhalation Anesthesia in a Patient With Xeroderma Pigmentosum: A Case Report

**DOI:** 10.5812/aapm.17880

**Published:** 2014-06-18

**Authors:** Mohammad Hajijafari, Mohammad Hossein Ziloochi, Mohammad Reza Fazel

**Affiliations:** 1 Department of Anesthesiology, Beheshti Hospital, Kashan University of Medical Sciences, Kashan, Iran; 2 School of Public Health, Tehran University of Medical Sciences, Tehran, Iran

**Keywords:** Xeroderma Pigmentosum, General Anesthesia, Sevoflurane

## Abstract

**Introduction:**

Xeroderma Pigmentosum (XP) is a rare autosomal recessive disease, which is defined by extreme sensitivity to sunlight and UV radiation and characterized by skin lesions and neuromuscular abnormalities. It is caused by a molecular defect in nucleotide excision repair genes. It has been reported that volatile anesthetics may cause genotoxic side effects or aggravation of the neurological signs. We report an XP patient with difficult intubation whose airway was controlled with Laryngeal Mask Airway (LMA) and was anesthetized with sevoflurane.

**Case Presentation:**

A 23-year-old woman, who had been a known case of XP since her childhood, was admitted to our hospital for excision of face mass (SCC) and skin graft surgery. Her airway examination revealed some anatomical and pathological abnormalities, including limitation of mouth opening, jaw protrusion, head extension, and class 4 of mallampati, all predicting difficult intubation. We chose general anesthesia with inhalation induction, LMA insertion and maintenance with sevoflurane without muscle relaxant. The surgery was completed uneventfully and the patient left the hospital the day after the surgery without any new complaint.

**Conclusions:**

We suggest that for XP patients with compromised air-way, sevoflurane (not all volatiles) may be preferred.

## 1. Introduction

Xeroderma Pigmentosum (XP) is a rare autosomal recessive disease, which is defined by extreme sensitivity to sunlight and ultraviolet radiation (1). It is caused by a molecular defect in nucleotide excision repair genes (2). Its incidence varies from 1 per 20,000 to 2.3 per 1 million live births; with higher incidence in North Africa and the Middle East (1-3). This disease appears with prolonged sunburn lesions, pigment changes (freckle-like) on the exposed skin and skin cancer, progressive neurological and neuromascular complications, and stiffness of the mouth and neck joints (2, 3). Little is known about how to manage the anesthesia of patients with XP (4-7). Halothane has been reported to have genotoxic side effects on the cells obtained from XP patients *in vitro* and may worsen the symptoms *in vivo*(7-9). No human data on this effect has been presented until now (10). Sevoflurane can exacerbate and deteriorate neurological complications in XP cases (8), yet there isn’t any recommendation to avoid the use of volatile anesthetics for these patients (11).This report describes general anesthesia with LMA in a patient with XP admitted for an excision of mass and skin graft surgery.

## 2. Case Presentation

**Figure 1.**
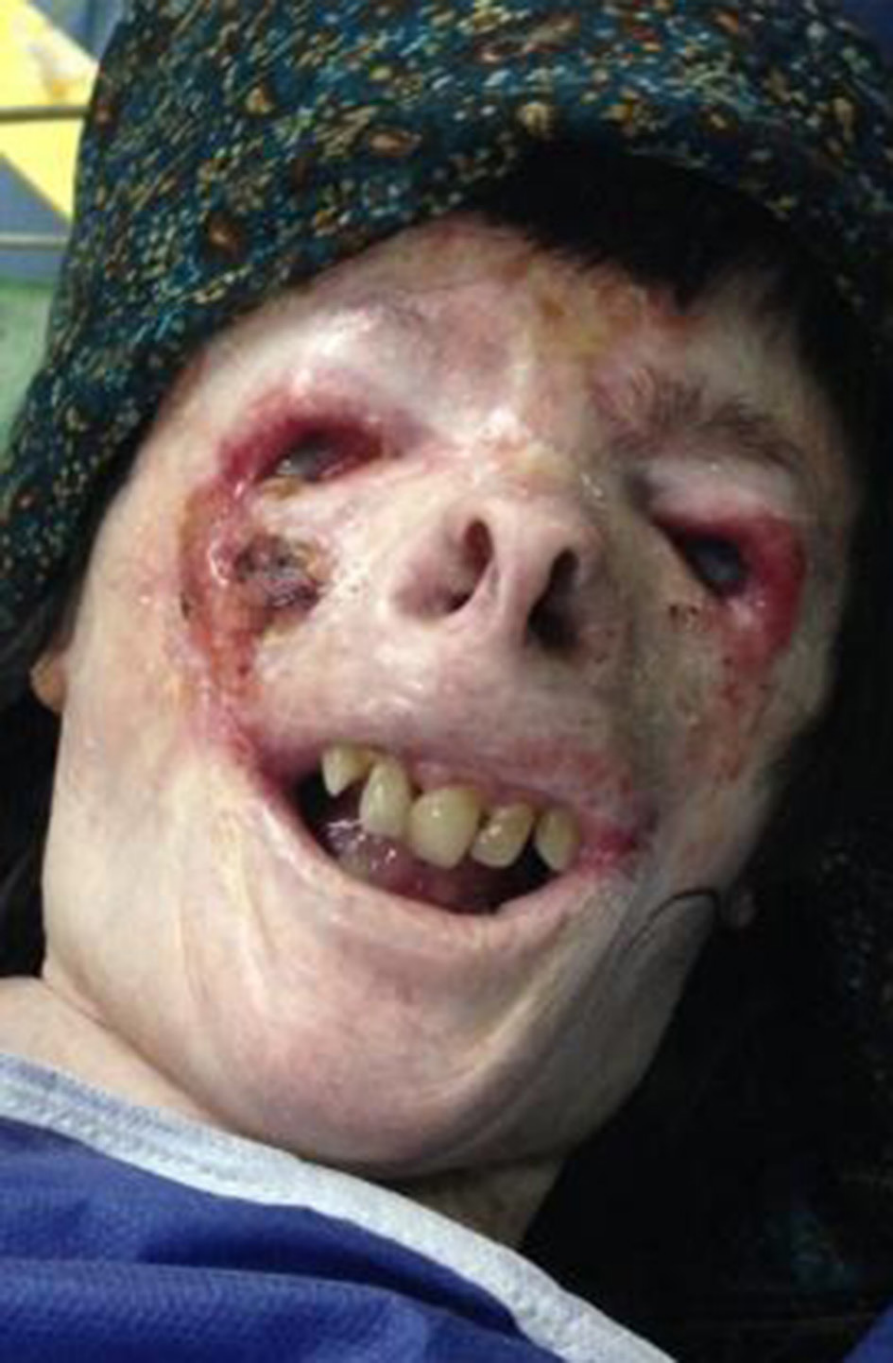
Abnormalities in the nose, mouth, and teeth of the patient; predicting difficult intubation

A 23-year-old woman, who had been a known case of XP since her childhood, was admitted to our hospital for excision of face mass and skin graft surgery. The pathology test of the lesion showed squamous cell carcinoma. Her airway examination revealed some anatomical and pathological abnormalities. The stiffness of her neck and jaw caused decreased motion and limited head extension and jaw protrusion. Her mouth opening was limited to < 20 mm; through this opening two relatively large upper incisor teeth were observed. Pharyngeal examination revealed score 4 mallampati. Also, we found *Pectuscarinatum* in her chest, a multiple erotic keratotic lesion and a deformity in her nose, and keratoconjunctivitis and corneal opacity in her eyes. All these findings predicted difficult intubation (Figure 1).

She had a short stature and weight of 28 Kg (BMI = 19), cachectic appearance with some deformities in the extremities. A neurological examination showed a minor decline in intelligence quotient (IQ). She was calm and cooperative but not completely oriented to time and space, with some decreased muscle strength and deep tendon reflexes. Her cardiovascular examination and paraclinical evaluation results were within the normal limit. General anesthesia was planned for the surgery. The patient was admitted to the operating room without any premedication to avoid unwanted sedation. All of the patient’s body was covered to protect it from UV and artificial lights in the operating room. We established standard monitoring including: pulse oximetry (SpO_2_), capnography (end tidal CO_2_), precordial cardioscopy and non-invasive blood pressure (NIBP). A peripheral venous on the arm was cannulated with a 22 G catheter. Once the preparations of anesthesia and surgery teams and the availability of difficult intubation equipment were ensured, the patient was cautiously pre-medicated intravenously with 25 µg of titrated fentanyl and 0.5 mg midazolam, to maintain spontaneous breathing. Then, general anesthesia was induced with inhalation of sevoflurane (manufactured by Aesica Queen borough, UK) 2.5% to 5% without using a neuromuscular agent while maintaining spontaneous breathing. After elimination of reflexes and reaching enough depth of anesthesia by bispectral index (BIS) count of about 55, an appropriate laryngeal mask (number 2.5) was inserted and floated; fixed when good lung ventilation was ensured. The surgeon infiltrated 5 ml of 2% lidocaine around the surgery site before starting the surgery. Anesthesia was maintained by 2% sevoflurane with a 50:50 mixture of oxygen and air. The procedure lasted 110 minutes without any complications. The patient’s non-invasive blood pressure (NIBP) reading was between 125 and 98 as maxima, and 81 and 6 mmHg as minima; her heart rate was between 75 and 10^5^ beats/minute, her SpO_2_ ranged from 97% to 100%, and her ETCO_2_ was between 33 and 37 mmHg. The surgery was completed uneventfully. After 10 minutes, when the patient was awake and obeyed to open her eyes, she was extubated. Postoperative analgesia was assured by bupivacaine infiltration around the wound at the end of the surgery and prescription of acetaminophen (as 325 mg-suppository) every four hours. The patient was discharged from the hospital one day after the surgery without any new complaints.

## 3. Discussion

Xeroderma Pigmentosum is a rare autosomal recessive disease, which is defined by extreme sensitivity to sunlight and ultraviolet UV radiation. Ultraviolet can destroy DNA of skin cells. Normally, there is a gene that repairs this damage, but in people with XP, it is not fixed due to molecular defects in the nucleotide excision repair mechanism (1, 2). Optimal anesthetic management of patients with XP is a dilemma. Many practitioners, being cautious about the probability of halogenated volatile gentoxicity, recommend total intravenous anesthesia (7, 8). For this patient, considering the probability of difficult intubation, general anesthesia with spontaneous breathing through inhalation induction and maintenance was chosen. Among anesthetic inhalation, sevoflurane has unique characteristic of having little metabolism with little metabolism and blood-gas partition coefficient, acceptable induction onset time and short awakening duration with a good hemodynamic stability. Propofol, also as an intravenous anesthetic has similar characteristics, yet it can induce apnea and was not the optimum choice for our patient, thus sevoflurane was used instead. Prolongation of recovery after using a muscle relaxant has been reported by some studies (7, 12). LMA was chosen for intubation because its insertion without using of muscle relaxant is more probable. Spontaneous ventilation was maintained during the insertion of LMA. The insertion was done without any difficulties, straining or hemodynamic instability. No hemodynamic instability, transient hypoxia, electrolyte disorder or new neurological findings were observed. Masuda in his study on XP patients recommended avoiding halothane because it may worsen the symptoms of XP (7). Reitz and Lanz also reported that halothane can induce an irreversible DNA change in lymphocytes of XP patients and a possible genotoxic side effect (9). In a report by Karabiyik et al. a transitory DNA damage was reported when normal human lymphocytes were exposed to sevoflurane and isoflurane *in vivo *(13).

Miyazaki in a case report presented an XP patient with a history of worsening transient neurological symptoms when a volatile substance was used as general anesthesia previously, whereas the perioperative course was uneventful with total intravenous anesthesia (TIVA) (8). On the other hand, Fjouji et al. reported similar findings regarding volatile agents and stated that volatile agents may deteriorate neurological status of XP patient, but they didn’t recommend using or not using this agent (11). Although some studies on animals presented information about volatile anesthetic-induced neurotoxicity, no human data for this effect is available (9). Shrestha (12) and Mulimani (14) reported several XP patients whom were successfully anesthetized with Propofol as TIVA. Oliveira (6) in a case report, described an anesthetic approach for an XP patient in which sevoflurane was used safely for maintenance of anesthesia and the patient had a good condition postoperatively. Now, volatile anesthetics are the most commonly used anesthetics worldwide, among them sevoflurane is often used for hemodynamically unstable patients, especially children. On the other hand, inhalation induction that is recommended for air way compromised patients was achieved with sevoflurane without serious complications. Sevoflurane is amongst preferred agents because of its ease of use and good condition perioperatively, even at high concentrations (6). Although sevoflurane is reported to be clinically safe in routine operations, its application for XP patients is not sufficiently practiced (11). Among IV anesthetics, dexmedetomidine (a potent alpha2-adrenoceptor agonist), may be preferred for these patients. Dexmedetomidine has sedative, analgesic and anxiolytic effects with minimal respiratory depression; however, its use is limited because of adverse events such as hypotension, bradycardia and nausea (15). Before recommending the avoidance of volatile anesthetics in XP patients, we suggest more experimental and clinical studies to explain a probable genotoxicity or neurotoxicity of volatile anesthetics. A patient with XP who is a candidate for surgery is at significant risks, such as worsening disorders, failure of intubation or mechanical ventilation and post anesthesia complications. Based on case reports the use of volatile anesthetics, because of their genotoxicity, may have some adverse effects. At present, there are no clear recommendations for the avoidance of volatile agents in anesthetic management of patients with XP. More clinical and experimental research is needed to confirm the sensitivity of XP patients to sevoflurane and other halogenated anesthetics. Thus in general conditions the TIVA approach and in specific conditions, like airway compromisation, sevoflurane (not all volatiles) may be preferred.
